# Rituximab-induced interstitial lung disease: five case reports

**DOI:** 10.3402/ecrj.v2.27178

**Published:** 2015-05-21

**Authors:** Matiuallah Naqibullah, Saher B. Shaker, Karen S. Bach, Elisabeth Bendstrup

**Affiliations:** 1 Department of Respiratory Medicine, Gentofte University Hospital, Copenhagen, Denmark; 2 Department of Radiology, Gentofte University Hospital, Copenhagen, Denmark; 3 Department of Respiratory Medicine and Allergy, Aarhus University Hospital, Aarhus, Denmark

**Keywords:** Interstitial pneumonitis, lymphoma, rituximab

## Abstract

Rituximab (RTX), a mouse/human chimeric anti-CD20 IgG1 monoclonal antibody has been effectively used as a single agent or in combination with chemotherapy regimen to treat lymphoma since 1997. In addition, it has been used to treat idiopathic thrombocytopenic purpura, systemic lupus erythematous, rheumatoid arthritis, and autoimmune hemolytic anemia. Recently, RTX has also been suggested for the treatment of certain connective tissue disease–related interstitial lung diseases (ILD) and hypersensitivity pneumonitis. Rare but serious pulmonary adverse reactions are reported. To raise awareness about this serious side effect of RTX treatment, as the indication for its use increases with time, we report five cases of probable RTX-ILD and discuss the current literature on this potentially lethal association.

Rituximab (RTX), a mouse/human chimeric anti-CD20 IgG1 monoclonal antibody, has been effectively used as a single agent or in combination with chemotherapy regimen to treat lymphoma since 1997 (1, 2). In addition, it has been used to treat idiopathic thrombocytopenic purpura (3), systemic lupus erythematosus, rheumatoid arthritis (4), and autoimmune hemolytic anemia (2). Recently, RTX has also been suggested for the treatment of certain connective tissue disease–related interstitial lung diseases (ILD) (5) and hypersensitivity pneumonitis (6).

The most common side effects of RTX are infusion-related and include fever, chills, and rigors. These symptoms are usually self-limited. During clinical trials, infusion-related reactions occurred in 9–15% of patients, and respiratory manifestations such as cough, bronchospasm, sinusitis, and rhinitis occurred in 30% of cases. Patients may rarely experience severe side effects such as anaphylactic shock and acute respiratory distress syndrome (7), fatal in 0.04–0.07%. Although the rate of delayed neutropenia is slightly increased, the immediate risk of pulmonary infections is not increased. Besides, there is no evidence of an increased frequency of tuberculosis in patients with lymphoma treated with RTX (8).

A few cases have drawn our attention to the risk of RTX-induced ILD (RTX-ILD) (9), which may develop shortly after the initiation of treatment and lead to shortness of breath and dry cough in the absence of signs and biochemical markers of infection. Decline in lung function with markedly reduced diffusion capacity and new radiological changes are invariably present and respiratory failure may occur.

To raise awareness about this serious side effect of RTX treatment, as the indication for its use increases with time, we report five cases of probable RTX-ILD and discuss the current literature on this potentially lethal association.

## Case reports

### Case 1

**Fig. 1:**
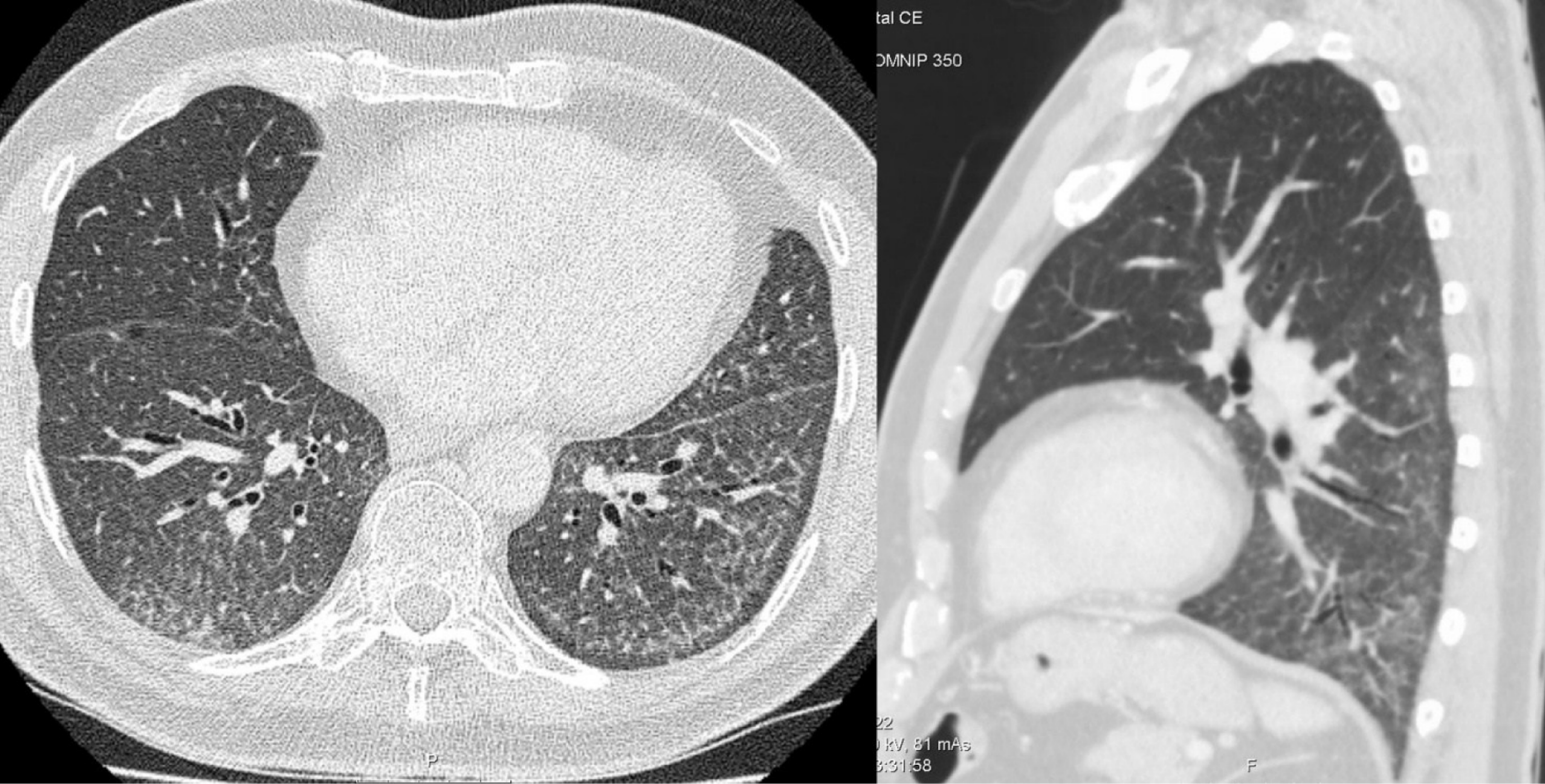
HRCT of the lungs showing diffuse ground glass opacity and fine reticulation with sparing of the immediate subpleural lung tissue on axial and sagittal planes.

A 68-year-old man with diffuse large B-cell lymphoma was referred with respiratory symptoms, 5 months after treatment with three cycles of RTX, cyclophosphamide, doxorubicin, vincristine, and prednisolone (R-CHOP). Prior to initiation of therapy, he had no respiratory symptoms and a normal computed tomography (CT) of the lungs. On referral, the patient complained of exertional dyspnea, cough, and fever. Physical examination revealed tachypnea, normal breath sounds, and normal saturation at rest. Lung function test (LFT) demonstrated a restrictive pattern with a reduced diffusion capacity for carbon monoxide (DLco 45%). Chest high-resolution (HR) CT demonstrated lung fields with diffuse ground glass opacities (GGO) and fine sub-pleural reticulation with sparing of the immediate sub-pleural lung, a pattern compatible with non-specific interstitial pneumonitis (NSIP) (Fig. 1). The diagnosis of RTX-ILD was made on clinical and radiological basis in multidisciplinary conference with no further need for invasive investigation. RTX therapy was immediately discontinued and the patient's condition stabilized without the need for steroid therapy. On follow-up, the patient's respiratory symptoms were completely recovered. His chest CT and DLco were normalized.

### Case 2

A 71-year-old man with stage IV follicular lymphoma was treated with two cycles of RTX and bendamustin with a 2-week interval. Prior to initiation of therapy, his clinical examination and chest X-ray were normal. He presented to our institution with fever, dyspnea, and dry cough, 1 month after the completion of the second treatment cycle. On physical examination, he had tachycardia, tachypnea, and hypoxemia requiring 2 L of oxygen. Spirometry demonstrated a restrictive pattern with very low diffusion capacity (DLco 26%).

**Fig. 2:**
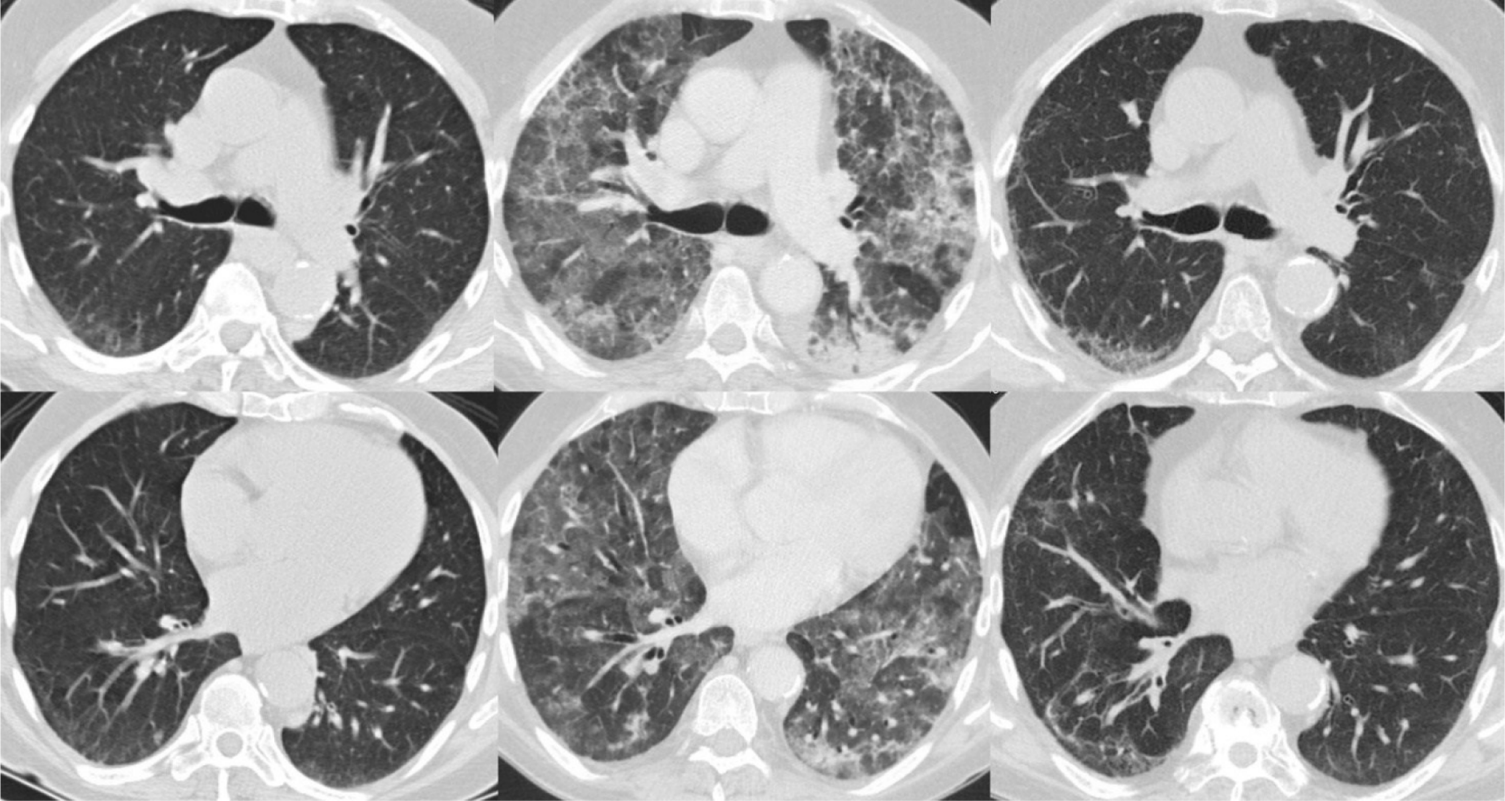
CT of the lungs at the level of carina and right inferior pulmonary vein prior to treatment with rituximab (left panel), during treatment with rituximab (middle panel), and after discontinuation of rituximab and initiation of steroid treatment.

HRCT of the chest after 1 month of completion of the second treatment cycle revealed diffuse fibrotic interstitial pneumonitis, with bilateral patchy consolidation and GGO, intervening with a normal lung. Sputum cultures were negative. The diagnosis of RTX-ILD was made on clinical and radiological bases in a multidisciplinary conference, which refrained from invasive procedures due to the high risk. The patient was started on 50-mg oral prednisolone daily with improvement in clinical symptoms. He was discharged from the hospital for planned control at the out-patient department. The symptoms and GGO improved considerably. Control HRCT of the chest after a month demonstrated reticulation with basal and peripheral predominance and relative sparing of the sub-pleural region, a pattern consistent with NSIP (Fig. 2).

A few months later, exertional dyspnea improved and DLco increased to 50%. HRCT revealed considerable regression of GGO, yet the chronic changes and subpleural reticulation persisted. The steroid therapy was withdrawn without deterioration of symptoms or lung function tests.

### Case 3

A 70-year-old man with Mantle-cell lymphoma was referred to our institution complaining of severe dyspnea and dry cough. Prior to initiation of RTX therapy, physical examination and chest CT were normal. The symptoms developed 1 week after the completion of the second cycle of R-CHOP therapy. He had severe hypoxemia and received high flow oxygen, and due to signs of infection broad-spectrum antibiotics were initiated. Spirometry revealed restrictive pattern with DLco at 26%. His condition worsened under admission; therefore, he was transferred to the intensive care unit, intubated, and mechanically ventilated. He was treated with high-dose intravenous steroid.

Chest CT demonstrated diffuse GGO with areas of consolidation. The diagnosis of RTX-ILD was considered possible, although severe infectious pneumonia was an obvious alternative. Bronchoalveolar lavage (BAL) was PCR positive for *Pneumocystis jirovecii* and he was switched to appropriate antibiotics. The last CT scan showed extensive consolidation and GGO intervening with normal lung and bilateral pleural effusion. After 2 weeks of intensive therapy, the patient died due to respiratory failure. No postmortem investigations were undertaken.

### Case 4

A 63-year-old man was treated with 20 series of extensive dose of radiotherapy for Hodgkin's lymphoma in 1969. The disease recurred in 1973, when he was treated with 12 series of chlormethine, vincristine, procarbazine, and prednisolone, followed by 52 series of platinum-containing chemotherapy in the following 2 years, which resulted in complete remission.

In 2010, the patient was diagnosed with large B-cell lymphoma and he received three series of R-CHOP followed by three further series of RTX alone. After three series of R-CHOP and a single RTX therapy, the patient developed severe and progressive exertional dyspnea. Until treatment with RTX was started, the patient had a stable but restrictively reduced spirometry and DLco (45%). RTX treatment resulted in deterioration of the diffusion capacity (DLco 21%).

HRCT revealed extensive partly confluent, poorly defined small nodules in a centrilobular pattern with characteristic sparing of the subpleural region, and mosaic attenuation. The radiological patterns resembled acute/subacute hypersensitivity pneumonitis. There were no signs of recurrence of lymphoma on the following PET scan.

RTX treatment was immediately discontinued and the patient was initially treated with 75 mg daily prednisolone with a rapid improvement of clinical symptoms and DLco, but the symptoms recurred shortly after reduction of prednisolone. He was thus switched to high-dose oral steroid pulse therapy, with little improvement but stabilization on follow-up. There was considerable improvement in DLco to 48%, which was the same level prior to initiation of RTX therapy.

### Case 5

A 63-year-old man was diagnosed with Mantle-cell lymphoma in 2009. He was treated with high doses of chemotherapy, followed by stem-cell transplantation with complete remission until January 2013, where the disease recurred in retroperitoneal and mediastinal lymph nodes. The patient was switched to weekly RTX for 4 weeks, followed by a combination of RTX and bendamustin. Prior to the initiation of RTX, his spirometry was normal and PET-CT revealed no lung parenchymal abnormalities.

The patient developed severe cough and exertional dyspnea immediately after treatment with the second series of RTX. Sputum cultures were negative. The patient completed the treatment regimen with four series. Body plethysmography showed restrictive reduction in lung function and low diffusion capacity (DLco 45%). HRCT of the lungs revealed diffuse GGO and nodular changes. BAL fluid analysis and transbronchial biopsies (TBB) revealed signs of eosinophilic inflammation with drug-induced pneumonitis as the possible underlying etiology.

The diagnosis of RTX-ILD was made on clinical, radiological, and histological bases. The patient was treated with prednisolone and follow-up revealed stability of symptoms and lung function parameters.

## Discussion

The introduction of biological agents into clinical practice has profoundly altered the clinical outcome in lymphatic malignancies as well as in different autoimmune diseases (10). At the same time, biological treatments have introduced new challenges, which require alertness and immediate reaction to discover toxicity, because these agents interfere with complex biological systems responsible for fundamental physiological functions (7, 10). There is a general consensus on the tolerability of biological agents, which have acceptable toxicity profile; nevertheless, some rare complications, including pneumonitis, may be underreported and require extra vigilance due to the life-threatening nature of these complications.

The incidence of RTX-ILD is unknown, but initial reports indicated very low incidence rates at 0.01–0.03% (10); however, much higher incidence rates were reported in post-marketing case series ranging from 3.7 to 10% (10, 11). This obvious difference in the incidence rate might be attributed to the difference in the target population between clinical trials and daily clinical practice. Besides, some cases of RTX-ILD might be regarded as lower respiratory tract infections, because of the overlap between the signs and symptoms of these complications. The definite causal relationship is difficult to prove, but chronological association together with the described clinical and radiological features make a probable diagnosis of RTX-ILD. Another challenge in the diagnosis is the use of supplementary immunosuppressive or chemotherapeutic agents particularly in the treatment of lymphoma. A high index of suspicion and careful search in the www.pneumtox.com database is extremely useful. This pragmatic approach has identified RTX as the common cause of lung injury in our case series.

The reported five cases in the current paper reveal the severe and potentially life-threatening pulmonary toxicity of RTX. The clinical and radiological changes develop within weeks of RTX treatment (12). The most common symptoms are dry cough, exertional dyspnea, and fever. Other less common and non-specific symptoms included fatigue, rigors, wheeze, hemoptysis, skin rash, and pleuritic chest pain. In 20% of cases, the patients were asymptomatic at the time of diagnosis with the disease being detected either by CT or LFT (10). Restrictive lung function and a moderate-to-severe reduction in DLco were invariably present in our patients.

CT of the lungs typically demonstrated diffuse or patchy bilateral consolidation and GGO, and in some cases, centrilobular nodules indicating the presence of alveolitis. In one case, bilateral pleural effusion were seen either due to RTX toxicity or co-existent infection. The fine reticulation seen in some cases might indicate the profibrotic nature of RTX lung toxicity and might explain the lack of normalization of LFT, when RTX is withdrawn. Our findings are consistent with the reported cases in the literature in which, alveolitis, fibrosis, alveolar hemorrhage, pleural effusions, and consolidation have been described (13, 14).

The pathogenic mechanism of RTX-ILD is very complex and yet to be elucidated. Several studies demonstrated that rapid lymphocytes lysis, complement activation (15), and TNF-α release occur after RTX infusion (16). In particular, TNF-α has been postulated as the main component in the pathogenesis of ILD because of its diverse proinflammatory effects by activating cytokines, inflammatory mediators, and angiogenic factors (17, 18). Lung biopsy is not usually performed in RTX-ILD, but pulmonary inflammation is a common feature in the reported cases. In our series, only one patient underwent TBB, showing eosinophilic inflammation. A recent systematic review (10) of the available studies showed histological patterns of organizing pneumonia, interstitial pneumonitis, desquamative interstitial pneumonia, diffuse alveolar damage, and usual interstitial pneumonia.

Routine control of lung function including spirometry and diffusion capacity can be justified before the initiation of RTX therapy and repeated if patients develop respiratory symptoms. The treatment of RTX-ILD follows the general principles of drug-induced pulmonary toxicity: 1) discontinuation of the offensive agent, 2) supportive treatment, and 3) corticosteroid treatment. Steroids can be considered in patients with moderate-to-severe dyspnea, respiratory failure, and severe decline in lung function, particularly the diffusion capacity. No recommendations can be made about the dose, route of administration, or duration of steroid therapy.

In conclusion, ILD is a rare but potentially fatal side effect of RTX therapy; therefore, physicians must maintain a high index of suspicion to recognize this side effect at early stages and prompt the discontinuation of RTX whenever possible, in order to prevent severe morbidity and mortality.
